# Supplementary material for: The causal role of circulating inflammatory markers in osteoporosis: a bidirectional Mendelian randomized study
Source: Front Immunol. 2024 Jul 18;15:1412298. doi: 10.3389/fimmu.2024.1412298 (PMC11291241; doi:10.3389/fimmu.2024.1412298)
Supplement: Supplementary file 1 [file DataSheet_1.zip › supplementary material/CIM-OSTEOPOROSIS /GCST90274843.csv_leave_one_out.pdf]

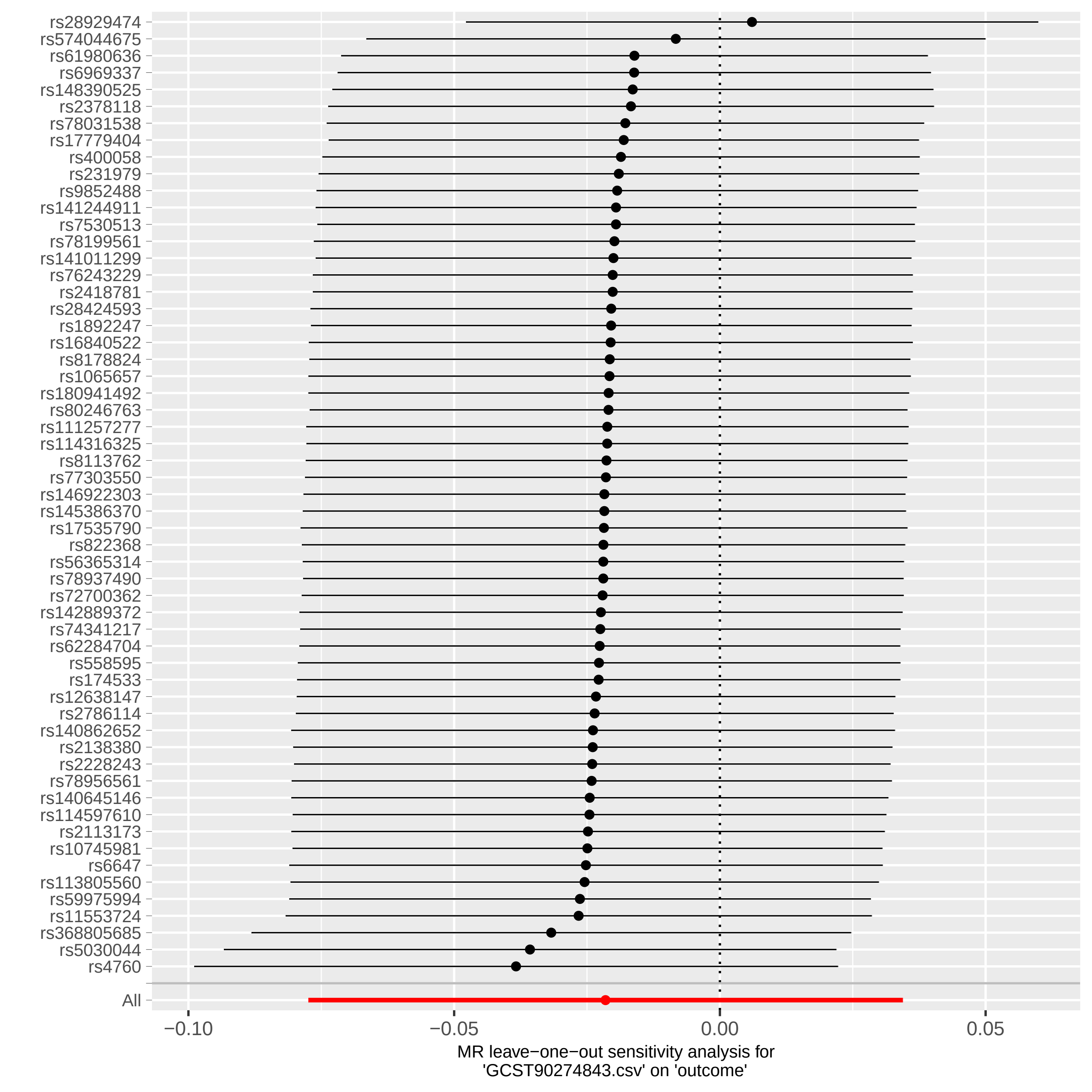
rs28929474
rs574044675
rs61980636
rs6969337
rs148390525
rs2378118
rs78031538
rs17779404
rs400058
rs231979
rs9852488
rs141244911
rs7530513
rs78199561
rs141011299
rs76243229
rs2418781
rs28424593
rs1892247
rs16840522
rs8178824
rs1065657
rs180941492
rs80246763
rs111257277
rs114316325
rs8113762
rs77303550
rs146922303
rs145386370
rs17535790
rs822368
rs56365314
rs78937490
rs72700362
rs142889372
rs74341217
rs62284704
rs558595
rs174533
rs12638147
rs2786114
rs140862652
rs2138380
rs2228243
rs78956561
rs140645146
rs114597610
rs2113173
rs10745981
rs6647
rs113805560
rs59975994
rs11553724
rs368805685
rs5030044
rs4760
All
−0.10
−0.05
0.00
0.05
MR leave−one−out sensitivity analysis for
'GCST90274843.csv' on 'outcome'
